# High-Intensity Focused Ultrasound Increases Facial Adipogenesis in a Swine Model via Modulation of Adipose-Derived Stem Cell Cilia

**DOI:** 10.3390/ijms25147648

**Published:** 2024-07-12

**Authors:** Kyung-A Byun, Hyoung Moon Kim, Seyeon Oh, Sosorburam Batsukh, Sangsu Lee, Myungjune Oh, Jeongwoo Lee, Ran Lee, Jae Woo Kim, Seung Min Oh, Jisun Kim, Geebum Kim, Hyun Jun Park, Hanbit Hong, Jehyuk Lee, Sang-Hyun An, Sung Suk Oh, Yeon-Seop Jung, Kuk Hui Son, Kyunghee Byun

**Affiliations:** 1 Department of Anatomy & Cell Biology, College of Medicine, Gachon University, Incheon 21936, Republic of Korea; 2 LIBON Inc., Incheon 22006, Republic of Korea; 3 Functional Cellular Networks Laboratory, Lee Gil Ya Cancer and Diabetes Institute, Gachon University, Incheon 21999, Republic of Korea; 4 Maylin Clinic, Goyang 10391, Republic of Korea; 5 Mirabel Clinic, Seoul 04596, Republic of Korea; 6 GangnamON Clinic, Seoul 06129, Republic of Korea; 7 ELEV Clinic, Seoul 06019, Republic of Korea; 8 Ezen Clinic, Cheonan 31090, Republic of Korea; 9 Lienjang Clinic, Seoul 04536, Republic of Korea; 10 MH Clinic, Seoul 06010, Republic of Korea; 11 Misogain Dermatology Clinic, Gimpo 10108, Republic of Korea; 12 Maylin Clinic the Cheongdam, Seoul 06091, Republic of Korea; 13 Lux Well Clinic, Cheongju 28424, Republic of Korea; 14 Doctorbom Clinic, Seoul 06614, Republic of Korea; 15 Preclinical Research Center, Daegu-Gyeongbuk Medical Innovation Foundation (K-MEDI Hub), Daegu 41061, Republic of Korea; 16 Medical Device Development Center, Daegu-Gyeongbuk Medical Innovation Foundation (K-MEDI Hub), Daegu 41061, Republic of Korea; 17 Department of Thoracic and Cardiovascular Surgery, Gachon University Gil Medical Center, Gachon University, Incheon 21565, Republic of Korea; 18 Department of Health Sciences and Technology, Gachon Advanced Institute for Health & Sciences and Technology (GAIHST), Gachon University, Incheon 21999, Republic of Korea

**Keywords:** adipogenesis, cilia, high-intensity focused ultrasound, zygomatic arch

## Abstract

Decreased medial cheek fat volume during aging leads to loss of a youthful facial shape. Increasing facial volume by methods such as adipose-derived stem cell (ASC) injection can produce facial rejuvenation. High-intensity focused ultrasound (HIFU) can increase adipogenesis in subcutaneous fat by modulating cilia on ASCs, which is accompanied by increased HSP70 and decreased NF-κB expression. Thus, we evaluated the effect of HIFU on increasing facial adipogenesis in swine (*n* = 2) via modulation of ASC cilia. Expression of *CD166*, an ASC marker, differed by subcutaneous adipose tissue location. *CD166* expression in the zygomatic arch (ZA) was significantly higher than that in the subcutaneous adipose tissue in the mandible or lateral temporal areas. HIFU was applied only on the right side of the face, which was compared with the left side, where HIFU was not applied, as a control. HIFU produced a significant increase in *HSP70* expression, decreased expression of *NF-κB* and a cilia disassembly factor (*AURKA*), and increased expression of a cilia increasing factor (*ARL13B*) and *PPARG* and *CEBPA*, which are the main regulators of adipogenesis. All of these changes were most prominent at the ZA. Facial adipose tissue thickness was also increased by HIFU. Adipose tissue volume, evaluated by magnetic resonance imaging, was increased by HIFU, most prominently in the ZA. In conclusion, HIFU increased ASC marker expression, accompanied by increased *HSP70* and decreased *NF-κB* expression. Additionally, changes in cilia disassembly and length and expression of adipogenesis were observed. These results suggest that HIFU could be used to increase facial volume by modulating adipogenesis.

## 1. Introduction

A youthful facial shape is formed by an even distribution of facial volume and provides a smooth transition following facial convexities [1]. During aging, all layers of the face between the skin and the facial bone, including fat pads, are changed [2]. Facial aging leads to a decreased volume of deep compartmental fat and downward migration of fat pads, which results in changes in the facial contour [3]. Moreover, a decreased volume in one area causes formation of skin folds in adjacent areas [4]. For example, decreased fat volume in the medial cheek leads to aggravation of nasolabial folds or under-eye folds [4]. Thus, maintenance of the midface fat volume is beneficial for a youthful facial appearance.

Various surgical face lift procedures, which lift the soft tissue and provide facial volume, have been performed for facial rejuvenation [5]. Less invasive procedures such as injection or transplantation of autologous fat have also been performed to increase facial volume [6]. However, autologous fat injection or transplantation is associated with problems related to partial absorption. The absorption rate has been reported to be in the range of 30–70% in 1 year [7].

Adult fat tissue contains adipose-derived stem cells (ASCs), which have the potential to differentiate into various cell lineages [8]. Because ASCs promote regeneration of various tissues, autologous fat acts as a dynamic filler that can promote regeneration of various facial tissues and produce volumetric effects [9,10,11]. The usual method of ASC isolation from fat tissues is enzymatic digestion via collagenase and centrifugation [12]. However, these procedures can affect ASC survival. Moreover, the ASC isolation process is time-consuming and expensive [13,14].

High-intensity focused ultrasound (HIFU) has been used for skin rejuvenation via tightening or lifting generated by thermal effect [15,16,17]. HIFU generates a selective microthermal lesion via accumulation of high-intensity focused ultrasound beam on the target area; however, HIFU causes minimal damage to surrounding tissue other than the target area [18].

Previously, our group reported that HIFU could increase adipogenesis in the subcutaneous fat of rat dorsal skin [19]. HIFU increased heat shock protein (HSP) 70, which was accompanied by decreased expression of nuclear factor kappa-light-chain-enhancer of activated B cells (NF-κB) and proinflammatory cytokines such as interleukin (IL)-6 and tumor necrosis factor-α (TNF-α) [19]. Moreover, HIFU decreased the expression of cilia disassembly-related factors such as aurora kinase A (AURKA) and histone deacetylase 6 and increased cilia assembly-related factors such as kinesin superfamily protein 3 (KIF3) and intraflagellar transport protein 88 (IFT88) in the ASCs of subcutaneous adipose tissue [19]. IL-6 and TNF-α have been reported to affect cilia length [19,20]. Additionally, primary cilia are involved in the inhibition of Wnt signaling [21]. Because the primary cilia length, which is affected by cilia assembly and disassembly processes, affects adipogenesis [22,23], HIFU may lead to changes in primary cilia of ASCs via increasing HSP70 and decreasing proinflammatory cytokines, leading to adipogenesis, which is eventually involved in increasing adipogenic differentiation of ASCs [19].

Bichat’s fat pad (buccal fat pad) consists of capsulated fat in the cheek and has four anatomical extensions, buccal, pterygoid, superficial, and deep temporal [24]. It is located between the anterior margin of the masseter and buccinator muscles and expands toward the mandibular ramus and laterally to the zygomatic arch (ZA) [24]. Because Bichat’s fat pad occupies the deep space of the face, it provides cheek volume. However, the volume of the fat pad changes during aging [24].

Bichat’s fat pad has been used as a fat source in facial fat transfer procedures [25]. Moreover, ASCs isolated from Bichat’s fat pad have been reported to be a suitable source for organ regeneration [26,27,28,29]. Mesenchymal stem cells (MSCs) from Bichat’s fat pad have a faster mitosis rate and greater number of perivascular elements than MSCs from abdominal adipose tissue, which suggests that MSCs from this fat pad have good regenerative potential [30].

Because Bichat’s fat pad has ASCs that show regenerative capacity and the volume of this fat pad affects the facial contour, it is expected that stimulation of ASCs in the buccal area by HIFU could lead to changes in adipogenesis, which will eventually affect the facial contour. We hypothesized that HIFU increases adipogenesis by changing the function of ASC cilia in the buccal area, which is accompanied by increased HSP70 and decreased NF-κB expression. Therefore, we evaluated the effect of HIFU on adipogenesis in the buccal area using a swine model.

## 2. Results

### 2.1. CD166 Expression Differs Depending on the Location of Subcutaneous Adipose Tissue

**Figure 1:**
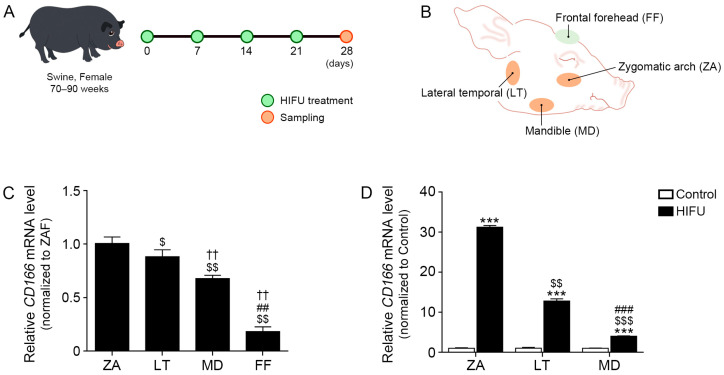
Regulation of ASC markers by HIFU treatment in various types of subcutaneous adipose tissue. (**A**) Schematic of swine used to assess HIFU regarding increases in subcutaneous adipose tissue thickness. (**B**) Schematic of various types of subcutaneous adipose tissue in swine. (**C**) The mRNA level of *CD166* in the ZA, LT, MD, and FF was measured by qRT-PCR. (**D**) The mRNA level of *CD166* in HIFU-treated areas including the ZA, LT, and MD was measured by qRT-PCR. Data are presented as the mean ± SD of three independent experiments. ***, *p* < 0.001, control vs. HIFU; $, *p* < 0.05, $$, *p* < 0.01, and $$$, *p* < 0.001, vs. ZA; ##, *p* < 0.01 and ###, *p* < 0.001, vs. LT; ††, *p* < 0.01, vs. MD (Mann–Whitney U test). ASC, adipose-derived stem cell; FF, frontal forehead; HIFU, high-intensity focused ultrasound; LT, lateral temporal area; MD, mandible; qRT-PCR, quantitative reverse transcription–polymerase chain reaction; ZA, zygomatic arch.

We evaluated whether expression of CD166, an ASC marker [31], in the subcutaneous adipose tissue differs depending on the location within the face using minipigs (Figure 1A,B). *CD166* expression in subcutaneous adipose tissue around the ZA was higher than that of the subcutaneous adipose tissue around the frontal forehead (FF), mandible (MD), or lateral temporal (LT) area (Figure 1C). Because we hypothesized that HIFU affects ASCs in the adipose tissue, we applied HIFU to three different areas, the ZA, LT, and MD, which differ in *CD166* expression, and compared the effect of HIFU on these areas. We did not apply HIFU to the FF area because of the low *CD166* expression in this area. *CD166* expression in the ZA, LT, and MD tissues where HIFU was applied was significantly higher than that of the control side. Increased *CD166* expression induced by HIFU was most prominent in the ZA (Figure 1D).

### 2.2. HIFU Increases HSP70 Expression and Decreases NF-κB Expression in Facial Subcutaneous Adipose Tissue

**Figure 2:**
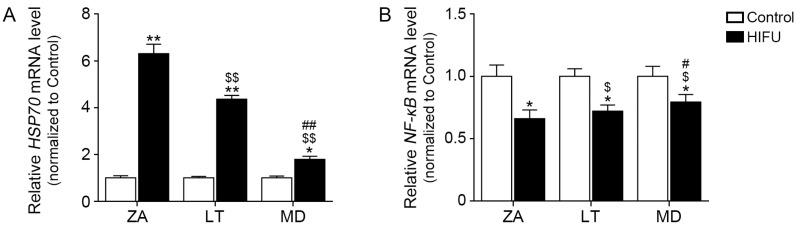
Regulation of *HSP70* and *NF-κB* by HIFU treatment in various types of subcutaneous adipose tissue. (**A**,**B**) The mRNA levels of *HSP70* and *NF-κB* in HIFU-treated ZA, LT, and MD tissues were measured by qRT-PCR. Data are presented as the mean ± SD of three independent experiments. *, *p* < 0.05 and **, *p* < 0.01, control vs. HIFU; $, *p* < 0.05 and $$, *p* < 0.01, vs. ZA; #, *p* < 0.05 and ##, *p* < 0.01, vs. LT (Mann–Whitney U test). HIFU, high-intensity focused ultrasound; HSP70, heat shock protein 70; LT, lateral temporal area; MD, mandible; NF-κB, nuclear factor kappa-light-chain-enhancer of activated B cells; qRT-PCR, quantitative reverse transcription–polymerase chain reaction; ZA, zygomatic arch.

HSP70 expression in the ZA, LT, and MD tissues where HIFU was applied was significantly higher than that of the control side. Increased HSP70 expression induced by HIFU was most prominent in the ZA (Figure 2A). Additionally, *NF-κB* expression in the ZA, LT, and MD tissues where HIFU was applied was significantly lower than that of the control side. Decreased *NF-κB* expression induced by HIFU was also most prominent in the ZA (Figure 2B).

### 2.3. HIFU Decreases Cilia Disassembly-Related Factors and Increases Cilia Length Increasing Factors in Facial Subcutaneous Adipose Tissue

**Figure 3:**
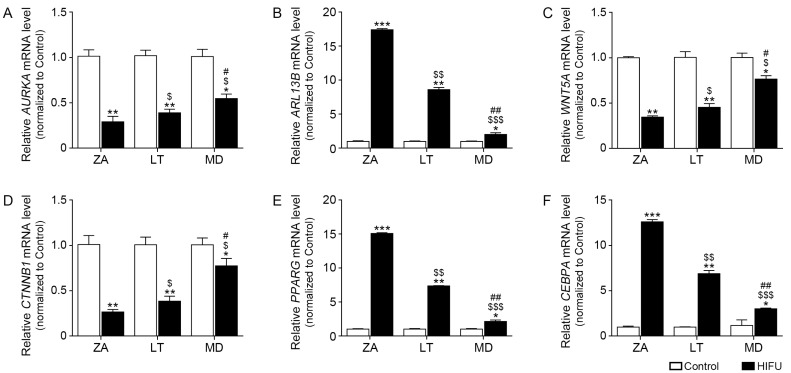
Regulation of cilia disassembly-related factors, cilia length increasing factors, and adipogenesis factors by HIFU treatment in various types of subcutaneous adipose tissue. (**A**,**B**) The mRNA levels of *AURKA* and *ARL13B* in HIFU-treated ZA, LT, and MD tissues were measured by qRT-PCR. (**C**,**D**) The mRNA levels of *WNT5A* and *CTNNB1* in HIFU-treated ZA, LT, and MD tissues were measured by qRT-PCR. (**E**,**F**) The mRNA levels of *PPARG* and *CEBPA* in HIFU-treated ZA, LT, and MD tissues were measured by qRT-PCR. Data are presented as the mean ± SD of three independent experiments. *, *p* < 0.05, **, *p* < 0.01, and ***, *p* < 0.001, control vs. HIFU; $, *p* < 0.05, $$, *p* < 0.01, and $$$, *p* < 0.001, vs. ZA; #, *p* < 0.05 and ##, *p* < 0.01, vs. LT (Mann–Whitney U test). ARL13B, ADP-ribosylation factor-like protein 13B; AURKA, aurora kinase A; CEBPA, CCAAT/enhancer binding protein α; CTNNB1, catenin beta 1; HIFU, high-intensity focused ultrasound; LT, lateral temporal area; MD, mandible; PPARG, peroxisome proliferator-activated receptor γ; qRT-PCR, quantitative reverse transcription–polymerase chain reaction; WNT5A, Wnt family member 5A; ZA, zygomatic arch.

*AURKA* expression in the ZA, LT, and MD tissues where HIFU was applied was significantly lower than that of the control side. Decreased *AURKA* expression induced by HIFU was also most prominent in the ZA (Figure 3A). Arl13b is a member of the Arf-like Ras superfamily of small GTPases that are involved in vesicle trafficking [32]. Moreover, Arl13b increases cilia length via upregulation of Forkhead box J1 (FoxJ1) and Regulatory factor X2 (Rfx2) [32]. *ARL13B* expression in the ZA, LT, and MD tissues where HIFU was applied was significantly higher than that of the control side. Increased *ARL13B* expression induced by HIFU was also most prominent in the ZA (Figure 3B).

### 2.4. HIFU Application Increases Adipogenesis Factors

*WNT5A* expression in the ZA, LT, and MD tissues where HIFU was applied was significantly lower than that of the control side. Decreased *WNT5A* expression induced by HIFU was also most prominent in the ZA (Figure 3C). Expression of Catenin beta 1 (*CTNNB1*) mRNA, which is an important gene that produces beta-catenin protein [33] was decreased by HIFU in the ZA, LT, and MD. Decreased *CTNNB1* expression induced by HIFU was also most prominent in the ZA (Figure 3D).

Peroxisome proliferator-activated receptor γ (*PPARG*) and CCAAT/enhancer binding protein α (*CEBPA*) are the main regulators of adipogenesis [34]. *PPARG* expression in the ZA, LT, and MD tissues where HIFU was applied was significantly higher than that of the control side (Figure 3E). *CEBPA* expression in the ZA, LT, and MD tissues where HIFU was applied was significantly higher than that of the control side (Figure 3F). The greatest difference in *PPARG* and *CEBPA* expression between the control side and the HIFU-applied side was shown in the ZA.

### 2.5. HIFU Increases the Thickness of Facial Adipose Tissue and Number of Adipocytes

**Figure 4:**
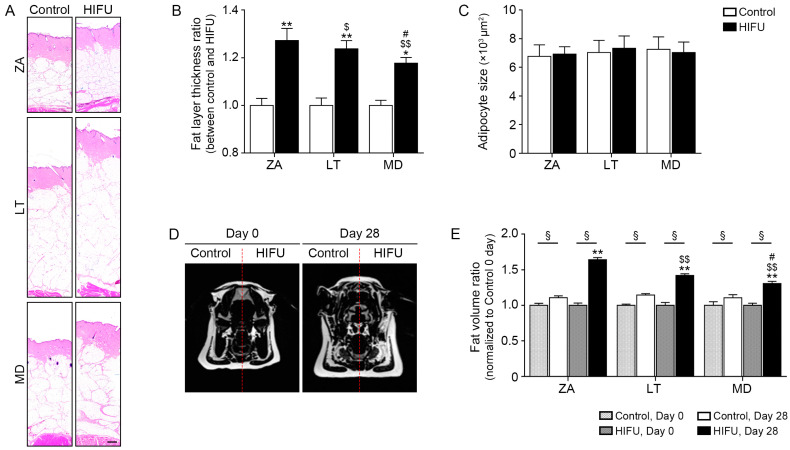
Regulation of adipogenesis by HIFU treatment in various types of subcutaneous adipose tissue. (**A**–**C**) The fat layer thickness and adipocyte size in HIFU-treated ZA, LT, and MD tissues were measured by hematoxylin and eosin staining. Scale bar = 1 mm (**D**) Two-dimensional cross-sectional left (control) and right (HIFU) images of magnetic resonance imaging (MRI) before (day 0) and 28 days after HIFU application (day 28). The red dashed line is an imaginary line to distinguish control and HIFU. (**E**) The fat volume in HIFU-treated ZA, LT, and MD tissues was measured by MRI. Data are presented as the mean ± SD of three independent experiments. *, *p* < 0.05 and **, *p* < 0.01, control vs. HIFU; $, *p* < 0.05 and $$, *p* < 0.01, vs. ZA; #, *p* < 0.05, vs. LT; §, *p* < 0.05, day 0 vs. day 28 (Mann–Whitney U test). HIFU, high-intensity focused ultrasound; LT, lateral temporal area; MD, mandible; ZA, zygomatic arch.

The thickness of subcutaneous adipose tissue in the ZA, LT, and MD tissues where HIFU was applied was significantly greater than that of the control side. The greatest difference in fat thickness between the control side and the HIFU-applied side was observed in the ZA (Figure 4A,B). The adipocyte sizes of the ZA, MD, and LT tissues where HIFU was applied were not different from those of the control side (Figure 4A,C).

Fat volume was evaluated by magnetic resonance imaging (MRI) before (day 0) and 28 days after HIFU application (day 28). The fat volume of the control side of the ZA, LT, and MD was increased 28 days after HIFU application compared with that on day 0. This increase might be induced by minipig growth over time. However, the fat volume of the HIFU-applied side of the ZA, LT, and MD was increased 28 days after HIFU application compared with that on day 0 and these increases were greater than those on the control side. The fat volume difference between day 0 and day 28 on the HIFU-applied side was greatest in the ZA (Figure 4D,E).

## 3. Discussion

This study demonstrated that HIFU increased facial subcutaneous adipose tissue thickness, which was associated with increased expression of an ASC marker (CD166) and master regulators of adipogenesis (PPAR-γ and C/EBPα). The effects of HIFU differed by subcutaneous adipose tissue location.

From the perspective of obesity control, reduction in adipogenesis has been thought to be beneficial, but from a regenerative medicine perspective, proper adipogenesis is required to regenerate soft tissue. Recently, adipose tissue regeneration was shown to be necessary to restore adipose tissue defects caused by burns or skin wound defects [35,36,37,38]. Adipose tissue regeneration has also attracted interest as an aesthetic procedure for facial rejuvenation [37,38].

A previous study from our group demonstrated that HIFU could modulate primary cilia of ASCs in subcutaneous adipose tissue, which eventually increased adipogenesis in the animal skin [19]. Thus, we hypothesized that HIFU could increase facial volume via stimulation of adipogenesis. In particular, HIFU could stimulate ASCs in situ, which differs from the effect of autologous fat transplantation. Because the autologous fat isolation process can negatively impact cell survival, we speculated that HIFU could be a more reasonable alternative to autologous fat transplantation.

Because we speculated that HIFU stimulates ASCs in facial adipose tissue, we evaluated whether the number of ASCs differed based on the adipose tissue location. Interestingly, CD166 expression was highest in the subcutaneous adipose tissue near the ZA among four measurement locations. In contrast, subcutaneous adipose tissue near the FF demonstrated the lowest CD166 expression. Although several studies have shown that Bichat’s fat pad can be a source of ASCs [26,27], studies showing differences in the number of ASCs by facial location are rare. Our study results suggested that even subcutaneous adipose tissue contains differing amounts of ASCs according to the tissue location. The results suggested that the effect of HIFU could differ by facial location because the number of ASCs differed by facial location. In our study, CD166 expression was increased by HIFU, and this increase differed by adipose tissue location. Adipose tissue near the ZA, where CD166 expression was highest, showed the most prominent increase after HIFU application.

Our previous study demonstrated that HIFU increased HSP70 and decreased NF-κB expression in adipose tissue [19]. Those changes were accompanied by decreased IL-6 and TNF-α expression [19]. Both IL-6 and TNF-α have been reported to be involved in primary cilia function via increasing cilia disassembly [39]. Similarly, our previous study demonstrated that decreased IL-6 and TNF-α expression was accompanied by decreases in cilia disassembly factors in adipose tissue [19].

Adipocytes are differentiated from MSCs, and their main role is storage of excessive energy [40,41]. Because excessive energy is stored as lipid droplets in mature adipocytes, both hypertrophy (increased size) and hyperplasia (increased number) of adipocytes could occur as responses to manage excessive energy [40,41]. Adipogenesis, which is a process by which adipocytes are formed from MSCs, is divided into two phases, determination and terminal differentiation [42,43,44,45]. In the determination phase, MSCs become committed adipocyte precursors (preadipocytes) and lose their potential to be differentiated into other cell lineages [42,43,44,45]. Various cell signaling pathways, such as Wnt, bone morphogenetic protein, insulin, leptin, and Hedgehog signaling, are involved in the adipocyte differentiation process. These signaling pathways modulate C/EBPs and PPAR-γ, which regulate the fate of adipogenic cells [40,42,46,47].

Primary cilia are involved in adipogenesis [40,42,46,47] and are prevalent in MSCs during adipogenesis [22]. KIF3a, a cilia assembly factor, is required during the early stage of adipogenic differentiation [48,49]. Additionally, reduction in ciliation by IFT88 knockdown in MSCs led to decreased adipogenesis [22]. In contrast, increases in primary cilia assembly led to adipogenesis by upregulation of C/EBPs and PPAR-γ [42,44,49,50]. The Wnt/β-catenin pathway is reported to inhibit C/EBP and PPAR-γ expression and inhibit adipogenic differentiation [51]. Moreover, primary cilia inhibit the Wnt5a/β-catenin pathway, which increases C/EBP and PPAR-γ expression [22].

In the present study, *HSP70* expression was increased by HIFU, and the increase differed by adipose tissue location. The HIFU-induced increase in *HSP70* expression was greatest in adipose tissue near the ZA, and this pattern was similar to that of expression changes in *CD166* induced by HIFU. The HIFU-induced decreases in *NF-κB* and the cilia disassembly factor *AURKA* were also greatest in adipose tissue near the ZA. In contrast, the cilia elongation factor *ARL13B* was most prominently increased by HIFU in the adipose tissue near the ZA. The forehead area, where *CD166* expression was not altered by HIFU, did not exhibit significant changes in *HSP70*, *NF-κB*, *AURKA*, or *ARL13B* expression after HIFU application. *WNT5A* and *CTNNB1* expression levels were most prominently decreased and expression levels of the adipogenesis markers *PPARG* and *CEBPA* were most prominently increased by HIFU in the adipose tissue near the ZA. Consistent with the pattern of increases in adipogenesis markers, the subcutaneous adipose tissue thickness near the ZA was most prominently increased by HIFU. The subcutaneous adipose tissue thickness could be increased by increasing both the adipocyte number and size. In contrast to increasing the number of adipocytes, increasing the adipocyte size leads to aggravation of insulin resistance and chronic inflammation, which results in adipose tissue dysfunction [52,53].

Dysfunctional adipose tissue secretes increased amounts of inflammatory factors, such as TNF-α, IL-6, and NF-κB, which induce chronic inflammation [54,55,56]. Our study showed that HIFU increased adipose tissue thickness without increasing adipocyte size. These results suggested that HIFU could increase subcutaneous adipose tissue thickness without increasing adipocyte dysfunction, including increasing inflammation.

There are several limitations in our study. The number of animals used in this study was small. It was known that the similarity of swine skin to human skin was higher than other animals, such as mice [57]; thus, swine were used in the study. Using swine was expensive, so a large number of pigs were not available in the study.

Considering the possible penetration depth of HIFU, it cannot be concluded that HIFU directly affected the Bichat fat pad. Moreover, we did not evaluate the cellular changes in Bichat’s fat pad in this study; therefore, it is not possible to determine whether HIFU affects this fat pad. Those are limitations of our study. In future studies, cellular changes in Bichat’s fat pad should be evaluated for revealing whether HIFU affects Bichat’s fat pad. However, the ASC distribution differed according to subcutaneous adipose tissue location, and factors involved in cilia length changes and adipogenesis were altered, consistent with the ASC distribution. Additionally, the subcutaneous adipose tissue thickness most prominently increased near the ZA, where the ASC distribution was highest. Therefore, this study confirmed that HIFU increases the ASC distribution, which is accompanied by a decrease in NF-κB and increases in factors related to cilia length and adipogenesis. These results suggest that HIFU can be used to increase adipogenesis of the face and can contribute to facial rejuvenation by increasing the midface volume because its effect is particularly prominent around the ZA.

## 4. Materials and Methods

### 4.1. HIFU System

A HIFU system (LinearZ, Jeisys Medical Inc., Seoul, Republic of Korea) was used in this study [19]. This transducer applied an energy of 0.1 J in DOT mode with a frequency of either 7 MHz at a focal depth of 4.5 mm or 2 MHz at a focal depth of 9 mm. To verify the focusing depth of the cartridge at 4.5 mm, 9 mm, a tissue-mimicking phantom was used. The phantom was prepared using the same method as described in the study by Kim et al. [58]. To assess the penetration depth of the HIFU-induced thermal lesion, a maximum energy of 3.0 J was applied at a single fixed point. This approach allowed us to accurately identify the thermal lesion without being influenced by other factors such as movement (Figure S1).

### 4.2. Animal Experiments and HIFU Application

All animal experiments were conducted in accordance with the guidelines of the institutional animal care and use committee and approved by the Daegu-Gyeongbuk Medical Innovation Foundation (approval number: KMEDI-23052401-00).

Female swine (40–50 kg, 70–90 weeks old) were obtained from CRONEX (Seoul, Republic of Korea). The minipigs were housed in cages with a 12 h light/dark cycle under a controlled temperature (22 ± 1 °C) and relative humidity (50 ± 10%). The animals had free access to reverse osmotic water, and the diet consisted of less than 2% of their body weight once a day during the entire test period (*n* = 2).

After acclimatization for 1 week, minipigs underwent MRI scans and then were subjected HIFU treatment with 200 J of total energy per week for 4 weeks (Figure 1A). For HIFU treatment of the location of fat identified by MRI scans, a 4.5 mm cartridge was used for the ZA, and a 9 mm cartridge was used for treatment of the MD and LT. HIFU treatment was performed while reconfirming the location and depth through sonography. The location of fat in ZA, MD, and LT can be reconfirmed through the HE results (Figure 4A). After 28 days, including four HIFU treatments, the minipigs were euthanized, and the ZA, LT, MD, and FF tissues were dissected (Figure 1B).

### 4.3. MRI Scan and Analysis

For the quantitative evaluation of the facial adipose tissue, each minipig was scanned by MRI once a week for 4 weeks using a 3T MRI scanner (Magnetom Skyra; Siemens, Erlangen, Germany) with a flexible 18-channel body coil. T1-weighted and fat images were obtained using 3D FLASH (Fast Low Angle Shot) and Dixon-VIBE (Volumetric Interpolated Breath-hold Examination using two-point Dixon fat-water separation) MR sequences, respectively. The parameters of the two sequences were as follows:

$$\begin{array}{l} \mathrm{TR}/\mathrm{TE}=12/5.63\;\mathrm{ms},\;\mathrm{FA}=9{}^{\circ},\;\mathrm{FOV}=480\times 480{\mathrm{mm}}^{2},\;\mathrm{slice}\;\mathrm{thickness}=1.5\mathrm{mm},\\ \mathrm{matrix}\;\mathrm{size}=320\times 320,\;\mathrm{number}\;\mathrm{of}\;\mathrm{slices}=88\;(@T1\text{-}\mathrm{weighted}), \end{array}$$

$$\begin{array}{l} \mathrm{TR}/\mathrm{TE}=4.21/1.31\;\mathrm{ms},\;\mathrm{FA}=25{}^{\circ},\;\mathrm{FOV}=480\times 480{\mathrm{mm}}^{2},\;\mathrm{slice}\;\mathrm{thickness}=1.5\mathrm{mm},\\ \mathrm{matrix}\;\mathrm{size}=320\times 320,\;\mathrm{number}\;\mathrm{of}\;\mathrm{slices}=144\;(@\mathrm{Dixon}\text{-}\mathrm{VIBE}), \end{array}$$

The generated fat volume was calculated using a 3D segmentation toolbox in SLICER (version 5.6.2, http://www.slicer.org, accessed on 5 April 2024) [59]. Regions of interest for the regions of HIFU treatment were segmented manually by two clinicians. Finally, volumes from the HIFU treatment site were compared to the volume of the contralateral region, quantitatively.

### 4.4. RNA Extraction and cDNA Synthesis

RNA extraction and cDNA synthesis from various adipose tissues were performed according to the instructions of the RNAiso reagent (TAKARA, Tokyo, Japan) and cDNA synthesis kit (TAKARA) [19]. The concentration of extracted RNA was measured using a Nanodrop spectrophotometer (Thermo Fisher Scientific, Waltham, MA, USA), and 1 μg of RNA was used for cDNA synthesis.

### 4.5. Quantitative Reverse Transcription–Polymerase Chain Reaction (qRT-PCR)

qRT-PCR was performed with a volume of 10 μL per well, including 2 μL of cDNA template, 5 μL of SYBR green premix (TAKARA), 0.4 μL each of the reverse and forward primers (Table S1), and 2.2 μL of TE buffer (Biosesang, Yongin, Republic of Korea), in a 384-well plate (Thermo Fisher Scientific). The process and melting curve analysis were performed using a QuantStudioTM 3 real-time PCR instrument (Thermo Fisher Scientific). The amplification procedure consisted of 10 min at 95 °C, 40 cycles of 15 s at 95 °C, 1 min at 60 °C, and 15 s at 95 °C. Afterward, melting analysis was performed from 95 °C to 60 °C at a rate of 0.075 °C/s. Gene expression levels were quantified using the comparative cycle threshold (ΔΔCT) method. The mRNA levels were normalized to those of the ACTB gene and compared with levels in the control.

### 4.6. Preparation of Paraffin-Embedded Tissue and Hematoxylin and Eosin Staining

The tissue was fixed in cold 4% paraformaldehyde (Sigma-Aldrich, St. Louis, MO, USA) for 48 h, placed in a cassette, and washed with distilled water. Then, the sample was placed in a tissue processor (Leica, Wetzlar, Germany), sequentially immersed in 95% and 99% ethanol (Duksan, Seoul, Republic of Korea), dehydrated, dipped in xylene (Duksan), and infiltrated with paraffin (Leica). Tissue blocks immersed in paraffin were processed into paraffin blocks using an embedding machine, sectioned to a thickness of 7 µm using a microtome (Leica), placed on a coated slide, incubated overnight at 60 °C, and attached to the slide.

For hematoxylin and eosin staining, section slides were deparaffinized, and rehydrated tissue slides were immersed in hematoxylin solution (KPNT, Cheongju, Republic of Korea) and then washed with tap water for 3 min. The tissue slides were then immersed in eosin solution (KPNT) for 1 min, washed with distilled water, dehydrated, and mounted using a DPX mounting solution (Sigma-Aldrich). Finally, the stained tissue was scanned using a slide scanner (Motic Scan Infinity 100; Motic, Beijing, China), and ten images were randomly captured. Quantitative analysis of proteins was performed using ImageJ software version 1.53s (NIH, Bethesda, MD, USA). Each group was compared with the control sample [60,61].

### 4.7. Statistical Analysis

All quantitative results are expressed as the mean ± SD of three experiments. The Kruskal–Wallis test was performed to compare groups, followed by the Mann–Whitney U test for post hoc comparisons. The results are expressed as the mean ± standard deviation (SD). All statistical analyses were performed using SPSS version 26 (IBM, Armonk, NY, USA). Statistical significance is indicated in each figure legend.

## 5. Conclusions

We showed that HIFU treatments of 0.1 J in DOT mode effectively increased expression of the ASC marker *CD166*, most prominently in the ZA. This increase was accompanied by increased *HSP70* expression and decreased *NF-κB* expression. The cilia length increasing factor *ARL13B* was increased, and the cilia disassembly factor *AURKA* was decreased. *PPARG* and *CEBPA* expression was increased by HIFU, which was accompanied by increased adipose tissue thickness. The changes induced by HIFU differed by facial adipose tissue location and were most prominent near the ZA. These results suggest that HIFU can be used to increase facial volume via modulation of adipogenesis.

## Data Availability

Data are contained within the article and Supplementary Materials.

## Supplementary Materials

The following supporting information can be downloaded at: https://www.mdpi.com/article/10.3390/ijms25147648/s1.
