# Supplementary material for: High-Intensity Focused Ultrasound Increases Facial Adipogenesis in a Swine Model via Modulation of Adipose-Derived Stem Cell Cilia
Source: Int J Mol Sci. 2024 Jul 12;25(14):7648. doi: 10.3390/ijms25147648 (PMC11277104; doi:10.3390/ijms25147648)
Supplement: Supplementary file 1 [file ijms-25-07648-s001.zip › ijms-3095142-supplementary.pdf]

International Journal of
Molecular Sciences

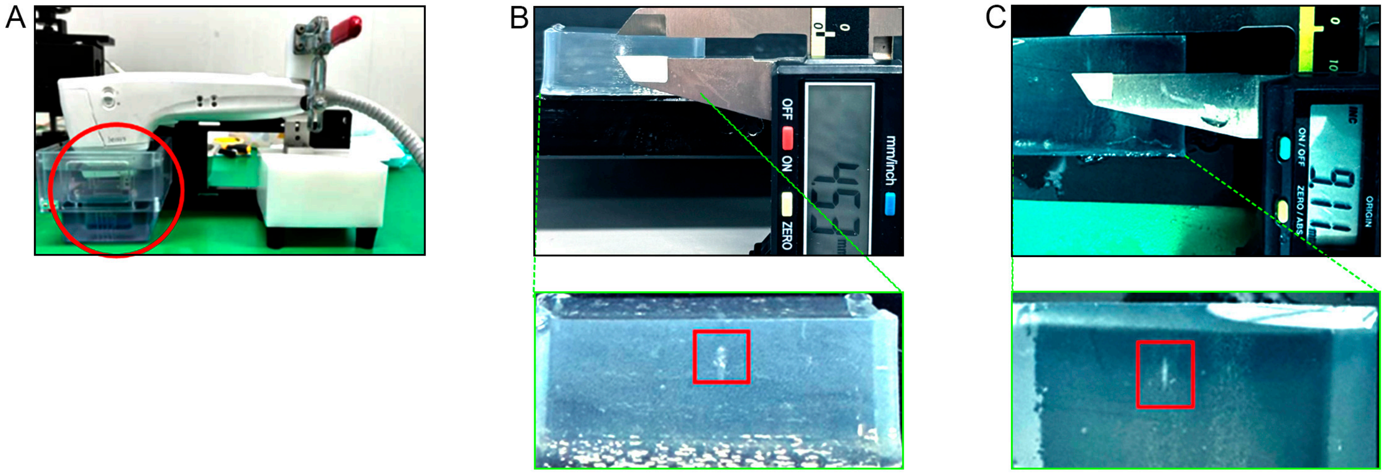

Figure S1. Cartridges were used for HIFU application at focal depths of 4.5 mm and 9 mm. (**A**) Schematic for cartridge identification at focal depths of 4.5 mm and 9 mm. (**B**) Depth of the tissue-mimicking phantom after applying a 4.5-mm cartridge. (**C**) Depth of the tissue-mimicking phantom after applying a 9-mm cartridge. HIFU, high-intensity focused ultrasound.

Table S1. List of primers for quantitative reverse–transcription polymerase chain reaction.

Gene	Primer Sequences	
CD166	Forward	5'-GAG CAG GTG ACA ATA CAA GTG C-3′
	Reverse	5'-GTT TCC CAG GCA TTT AAG AGT G-3′
HSP70	Forward	5'-ACC ACG CTA ATC CAG AGG AAT-3′
	Reverse	5'-AGG ACT CCA GGT TGG TTG TCT-3′
NF-κB	Forward	5'-CTA CCC TGG CAC AGA AAT TAG G-3′
	Reverse	5'-ATG AGA GTT TTG GAA GGA GCA G-3′
AURKA	Forward	5'-TGA GCA TCA GCT GAG AAG AGA A-3′
	Reverse	5'-ACT CTG GTA GCG TCA TGG AAA T-3′
ARL13B	Forward	5'-AGA CAC AAC AGA ACA ACG TGC T-3′
	Reverse	5'-TCT CTT TCC TCG CGT AAT TTT C-3′
WNT5A	Forward	5'-TAT GCC ACT TGT ATC AGG ACC A-3′
	Reverse	5'-TGA TAC TGG CAT TCT TTG ATG C-3′
CTNNB1	Forward	5'-TGT GCA GCT GGA ATT CTT TCT A-3′
	Reverse	5'- GAG CCT CTA TAC CAC CCA CTT G-3′
PPARG	Forward	5'-GCA TTT CCA CTC CAC ACT ATG A-3′
	Reverse	5'-CAC TTT GGT AGT CCT GGA GCT T-3′
CEBPA	Forward	5'-TGC TCA GAT ACT TGC CAA AAT G-3′
	Reverse	5'-CCA AAA CCA AAA GGA AAG ACA G-3′
ACTB	Forward	5'-ACC GAC TAC CTC ATG AAG ATC C-3′
	Reverse	5'-CGT AGC ACA GCT TCT CCT TGA T-3′
